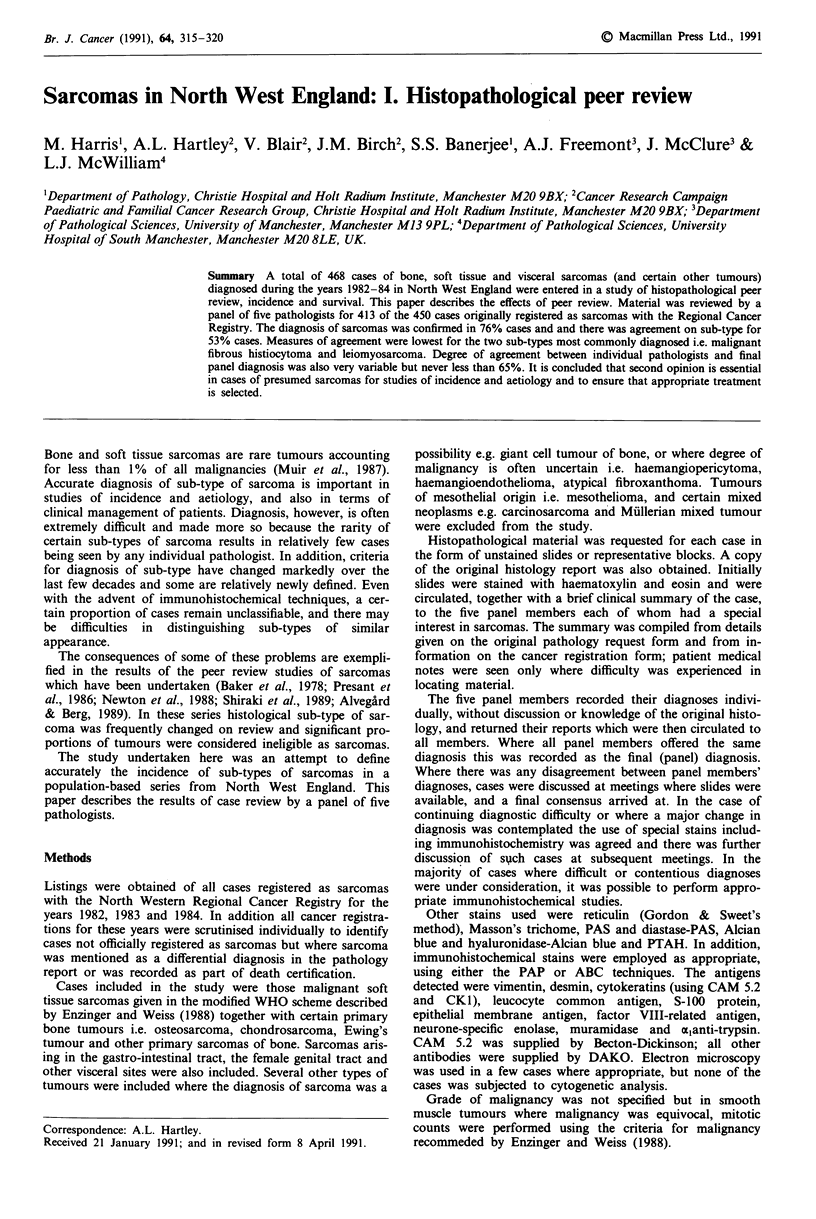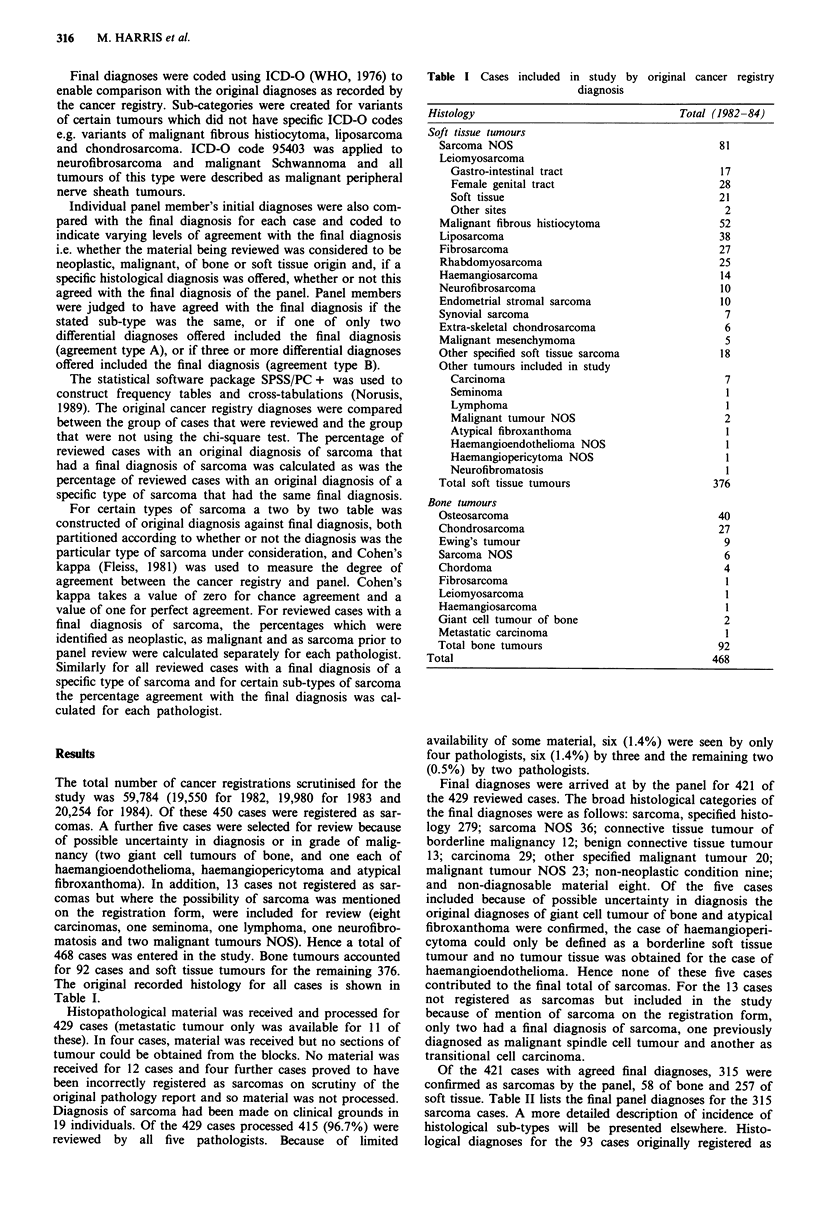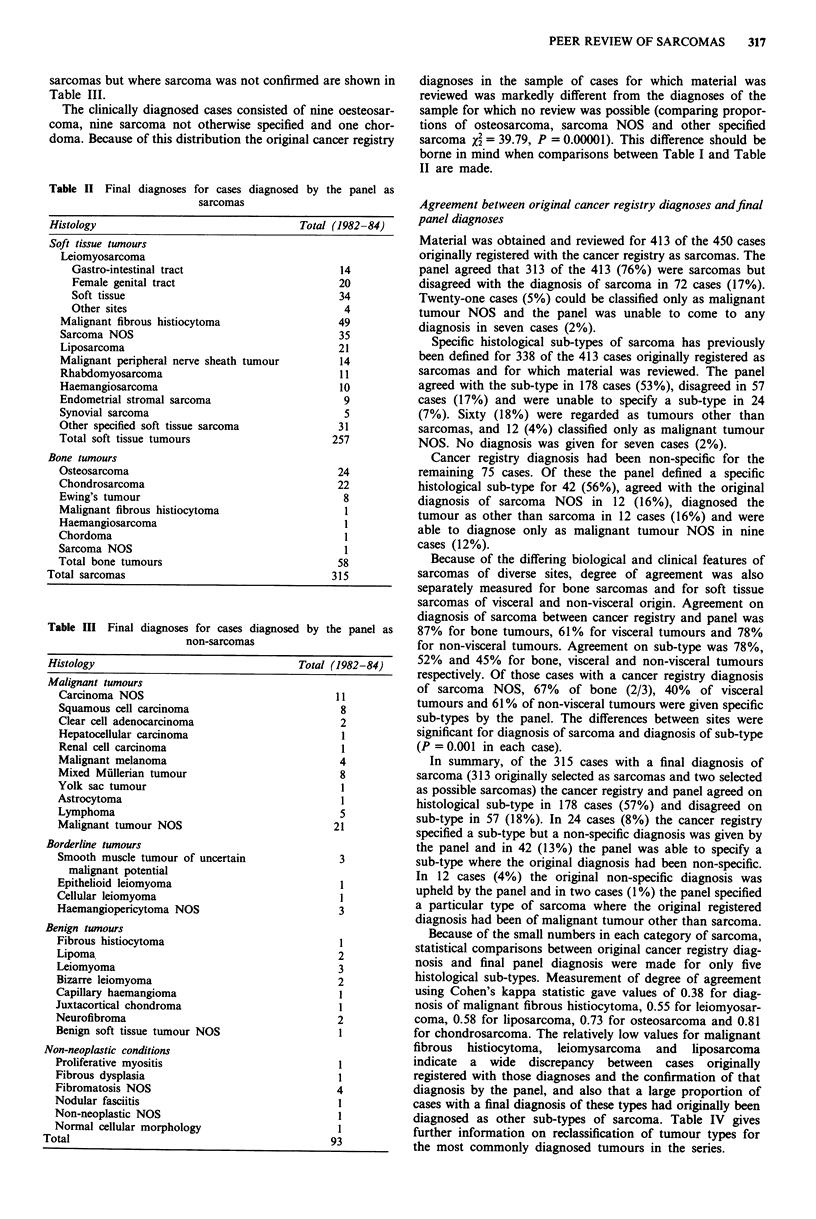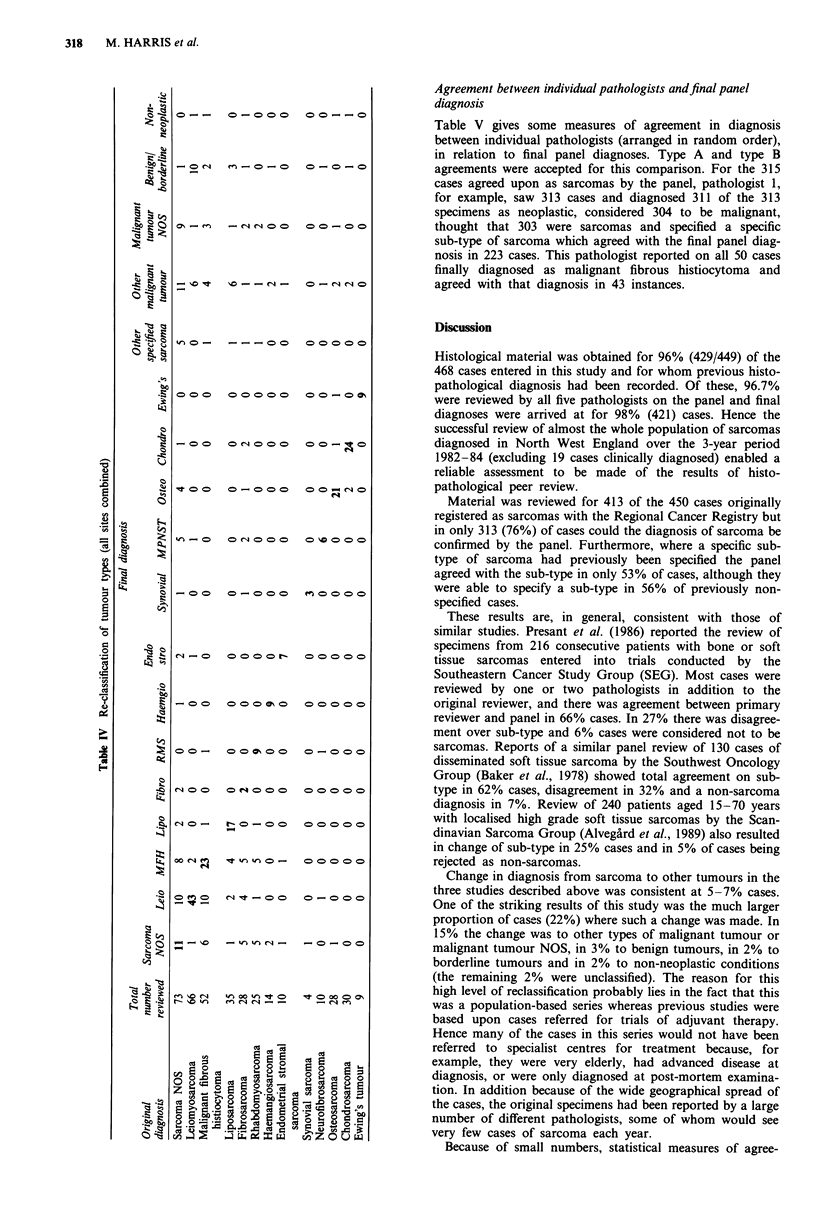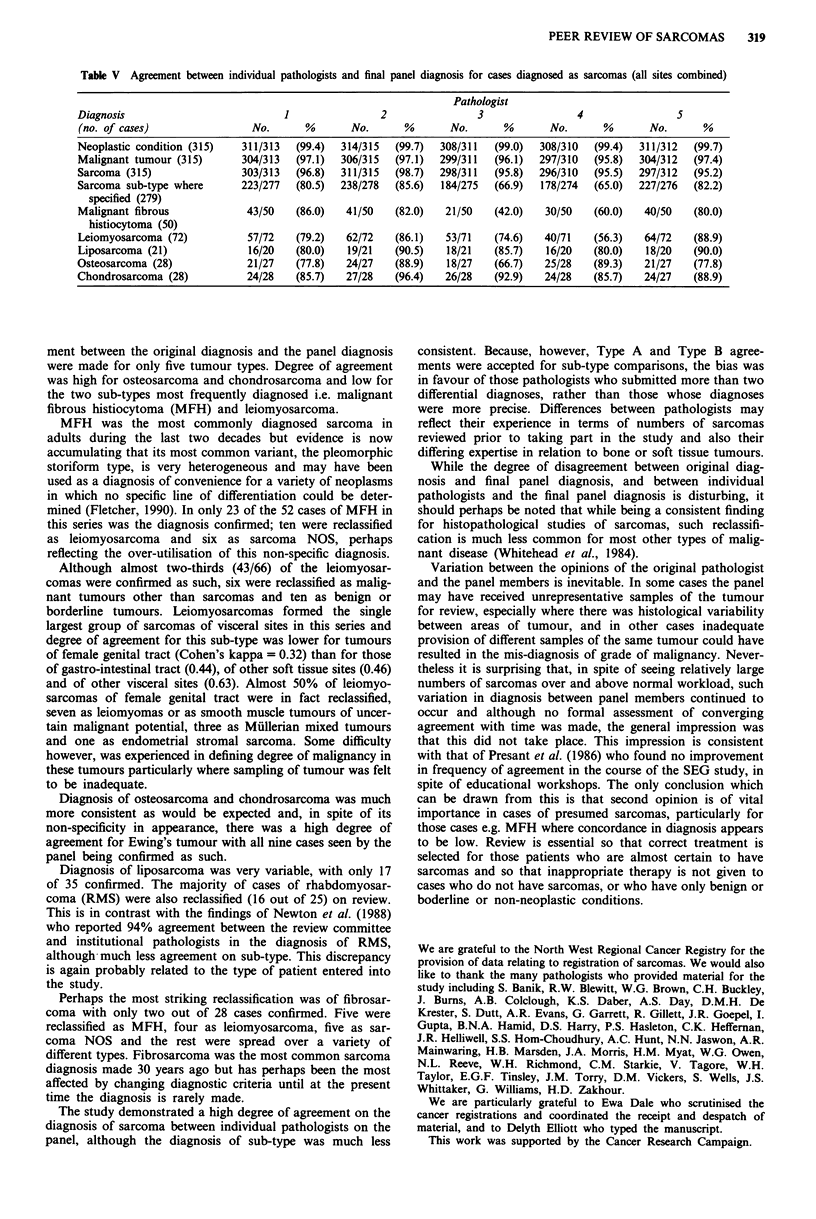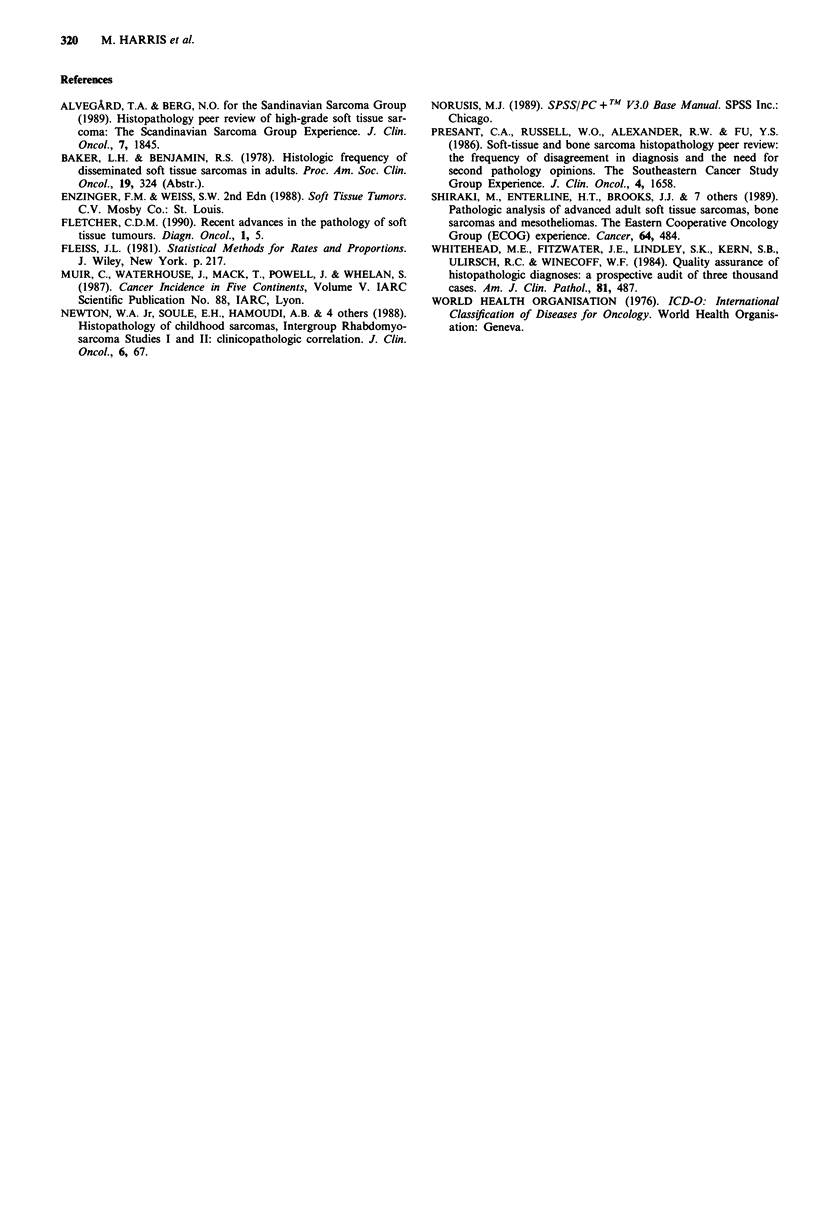# Sarcomas in north west England: I. Histopathological peer review.

**DOI:** 10.1038/bjc.1991.298

**Published:** 1991-08

**Authors:** M. Harris, A. L. Hartley, V. Blair, J. M. Birch, S. S. Banerjee, A. J. Freemont, J. McClure, L. J. McWilliam

**Affiliations:** Department of Pathology, Christie Hospital and Holt Radium Institute, Manchester, UK.

## Abstract

A total of 468 cases of bone, soft tissue and visceral sarcomas (and certain other tumours) diagnosed during the years 1982-84 in North West England were entered in a study of histopathological peer review, incidence and survival. This paper describes the effects of peer review. Material was reviewed by a panel of five pathologists for 413 of the 450 cases originally registered as sarcomas with the Regional Cancer Registry. The diagnosis of sarcomas was confirmed in 76% cases and and there was agreement on sub-type for 53% cases. Measures of agreement were lowest for the two sub-types most commonly diagnosed i.e. malignant fibrous histiocytoma and leiomyosarcoma. Degree of agreement between individual pathologists and final panel diagnosis was also very variable but never less than 65%. It is concluded that second opinion is essential in cases of presumed sarcomas for studies of incidence and aetiology and to ensure that appropriate treatment is selected.


*Br. J. Cancer* (1991), **64**, 315–320 © Macmillan Press Ltd., 1991

### Sarcomas in North West England: I. Histopathological peer review

M. Harris[1], A.L. Hartley[2], V. Blair[2], J.M. Birch[2], S.S. Banerjee[1], A.J. Freemont[3], J. McClure[3] & L.J. McWilliam[4]

[1]*Department of Pathology, Christie Hospital and Holt Radium Institute, Manchester M20 9BX;* [2]*Cancer Research Campaign Paediatric and Familial Cancer Research Group, Christie Hospital and Holt Radium Institute, Manchester M20 9BX;* [3]*Department of Pathological Sciences, University of Manchester, Manchester M13 9PL;* [4]*Department of Pathological Sciences, University Hospital of South Manchester, Manchester M20 8LE, UK.*

**Summary** A total of 468 cases of bone, soft tissue and visceral sarcomas (and certain other tumours) diagnosed during the years 1982–84 in North West England were entered in a study of histopathological peer review, incidence and survival. This paper describes the effects of peer review. Material was reviewed by a panel of five pathologists for 413 of the 450 cases originally registered as sarcomas with the Regional Cancer Registry. The diagnosis of sarcomas was confirmed in 76% cases and and there was agreement on sub-type for 53% cases. Measures of agreement were lowest for the two sub-types most commonly diagnosed i.e. malignant fibrous histiocytoma and leiomyosarcoma. Degree of agreement between individual pathologists and final panel diagnosis was also very variable but never less than 65%. It is concluded that second opinion is essential in cases of presumed sarcomas for studies of incidence and aetiology and to ensure that appropriate treatment is selected.

Bone and soft tissue sarcomas are rare tumours accounting for less than 1% of all malignancies (Muir *et al.*, 1987). Accurate diagnosis of sub-type of sarcoma is important in studies of incidence and aetiology, and also in terms of clinical management of patients. Diagnosis, however, is often extremely difficult and made more so because the rarity of certain sub-types of sarcoma results in relatively few cases being seen by any individual pathologist. In addition, criteria for diagnosis of sub-type have changed markedly over the last few decades and some are relatively newly defined. Even with the advent of immunohistochemical techniques, a certain proportion of cases remain unclassifiable, and there may be difficulties in distinguishing sub-types of similar appearance.

The consequences of some of these problems are exemplified in the results of the peer review studies of sarcomas which have been undertaken (Baker *et al.*, 1978; Presant *et al.*, 1986; Newton *et al.*, 1988; Shiraki *et al.*, 1989; Alvegård & Berg, 1989). In these series histological sub-type of sarcoma was frequently changed on review and significant proportions of tumours were considered ineligible as sarcomas.

The study undertaken here was an attempt to define accurately the incidence of sub-types of sarcomas in a population-based series from North West England. This paper describes the results of case review by a panel of five pathologists.

### Methods

Listings were obtained of all cases registered as sarcomas with the North Western Regional Cancer Registry for the years 1982, 1983 and 1984. In addition all cancer registrations for these years were scrutinised individually to identify cases not officially registered as sarcomas but where sarcoma was mentioned as a differential diagnosis in the pathology report or was recorded as part of death certification.

Cases included in the study were those malignant soft tissue sarcomas given in the modified WHO scheme described by Enzinger and Weiss (1988) together with certain primary bone tumours i.e. osteosarcoma, chondrosarcoma, Ewing's tumour and other primary sarcomas of bone. Sarcomas arising in the gastro-intestinal tract, the female genital tract and other visceral sites were also included. Several other types of tumours were included where the diagnosis of sarcoma was a possibility e.g. giant cell tumour of bone, or where degree of malignancy is often uncertain i.e. haemangiopericytoma, haemangioendothelioma, atypical fibroxanthoma. Tumours of mesothelial origin i.e. mesothelioma, and certain mixed neoplasms e.g. carcinosarcoma and Müllerian mixed tumour were excluded from the study.

Histopathological material was requested for each case in the form of unstained slides or representative blocks. A copy of the original histology report was also obtained. Initially slides were stained with haematoxylin and eosin and were circulated, together with a brief clinical summary of the case, to the five panel members each of whom had a special interest in sarcomas. The summary was compiled from details given on the original pathology request form and from information on the cancer registration form; patient medical notes were seen only where difficulty was experienced in locating material.

The five panel members recorded their diagnoses individually, without discussion or knowledge of the original histology, and returned their reports which were then circulated to all members. Where all panel members offered the same diagnosis this was recorded as the final (panel) diagnosis. Where there was any disagreement between panel members' diagnoses, cases were discussed at meetings where slides were available, and a final consensus arrived at. In the case of continuing diagnostic difficulty or where a major change in diagnosis was contemplated the use of special stains including immunohistochemistry was agreed and there was further discussion of such cases at subsequent meetings. In the majority of cases where difficult or contentious diagnoses were under consideration, it was possible to perform appropriate immunohistochemical studies.

Other stains used were reticulin (Gordon & Sweet's method), Masson's trichome, PAS and diastase-PAS, Alcian blue and hyaluronidase-Alcian blue and PTAH. In addition, immunohistochemical stains were employed as appropriate, using either the PAP or ABC techniques. The antigens detected were vimentin, desmin, cytokeratins (using CAM 5.2 and CK1), leucocyte common antigen, S-100 protein, epithelial membrane antigen, factor VIII-related antigen, neurone-specific enolase, muramidase and $\alpha_1$anti-trypsin. CAM 5.2 was supplied by Becton-Dickinson; all other antibodies were supplied by DAKO. Electron microscopy was used in a few cases where appropriate, but none of the cases was subjected to cytogenetic analysis.

Grade of malignancy was not specified but in smooth muscle tumours where malignancy was equivocal, mitotic counts were performed using the criteria for malignancy recommeded by Enzinger and Weiss (1988).

Correspondence: A.L. Hartley.
Received 21 January 1991; and in revised form 8 April 1991.

Final diagnoses were coded using ICD-O (WHO, 1976) to enable comparison with the original diagnoses as recorded by the cancer registry. Sub-categories were created for variants of certain tumours which did not have specific ICD-O codes e.g. variants of malignant fibrous histiocytoma, liposarcoma and chondrosarcoma. ICD-O code 95403 was applied to neurofibrosarcoma and malignant Schwannoma and all tumours of this type were described as malignant peripheral nerve sheath tumours.

Individual panel member's initial diagnoses were also compared with the final diagnosis for each case and coded to indicate varying levels of agreement with the final diagnosis i.e. whether the material being reviewed was considered to be neoplastic, malignant, of bone or soft tissue origin and, if a specific histological diagnosis was offered, whether or not this agreed with the final diagnosis of the panel. Panel members were judged to have agreed with the final diagnosis if the stated sub-type was the same, or if one of only two differential diagnoses offered included the final diagnosis (agreement type A), or if three or more differential diagnoses offered included the final diagnosis (agreement type B).

The statistical software package SPSS/PC + was used to construct frequency tables and cross-tabulations (Norusis, 1989). The original cancer registry diagnoses were compared between the group of cases that were reviewed and the group that were not using the chi-square test. The percentage of reviewed cases with an original diagnosis of sarcoma that had a final diagnosis of sarcoma was calculated as was the percentage of reviewed cases with an original diagnosis of a specific type of sarcoma that had the same final diagnosis.

For certain types of sarcoma a two by two table was constructed of original diagnosis against final diagnosis, both partitioned according to whether or not the diagnosis was the particular type of sarcoma under consideration, and Cohen's kappa (Fleiss, 1981) was used to measure the degree of agreement between the cancer registry and panel. Cohen's kappa takes a value of zero for chance agreement and a value of one for perfect agreement. For reviewed cases with a final diagnosis of sarcoma, the percentages which were identified as neoplastic, as malignant and as sarcoma prior to panel review were calculated separately for each pathologist. Similarly for all reviewed cases with a final diagnosis of a specific type of sarcoma and for certain sub-types of sarcoma the percentage agreement with the final diagnosis was calculated for each pathologist.

**Table I** Cases included in study by original cancer registry diagnosis

| *Histology* | *Total (1982–84)* |
|---|---|
| *Soft tissue tumours* | |
| Sarcoma NOS | 81 |
| Leiomyosarcoma | |
| Gastro-intestinal tract | 17 |
| Female genital tract | 28 |
| Soft tissue | 21 |
| Other sites | 2 |
| Malignant fibrous histiocytoma | 52 |
| Liposarcoma | 38 |
| Fibrosarcoma | 27 |
| Rhabdomyosarcoma | 25 |
| Haemangiosarcoma | 14 |
| Neurofibrosarcoma | 10 |
| Endometrial stromal sarcoma | 10 |
| Synovial sarcoma | 7 |
| Extra-skeletal chondrosarcoma | 6 |
| Malignant mesenchymoma | 5 |
| Other specified soft tissue sarcoma | 18 |
| Other tumours included in study | |
| Carcinoma | 7 |
| Seminoma | 1 |
| Lymphoma | 1 |
| Malignant tumour NOS | 2 |
| Atypical fibroxanthoma | 1 |
| Haemangioendothelioma NOS | 1 |
| Haemangiopericytoma NOS | 1 |
| Neurofibromatosis | 1 |
| Total soft tissue tumours | 376 |
| *Bone tumours* | |
| Osteosarcoma | 40 |
| Chondrosarcoma | 27 |
| Ewing's tumour | 9 |
| Sarcoma NOS | 6 |
| Chordoma | 4 |
| Fibrosarcoma | 1 |
| Leiomyosarcoma | 1 |
| Haemangiosarcoma | 1 |
| Giant cell tumour of bone | 2 |
| Metastatic carcinoma | 1 |
| Total bone tumours | 92 |
| Total | 468 |

### Results

The total number of cancer registrations scrutinised for the study was 59,784 (19,550 for 1982, 19,980 for 1983 and 20,254 for 1984). Of these 450 cases were registered as sarcomas. A further five cases were selected for review because of possible uncertainty in diagnosis or in grade of malignancy (two giant cell tumours of bone, and one each of haemangioendothelioma, haemangiopericytoma and atypical fibroxanthoma). In addition, 13 cases not registered as sarcomas but where the possibility of sarcoma was mentioned on the registration form, were included for review (eight carcinomas, one seminoma, one lymphoma, one neurofibromatosis and two malignant tumours NOS). Hence a total of 468 cases was entered in the study. Bone tumours accounted for 92 cases and soft tissue tumours for the remaining 376. The original recorded histology for all cases is shown in Table I.

Histopathological material was received and processed for 429 cases (metastatic tumour only was available for 11 of these). In four cases, material was received but no sections of tumour could be obtained from the blocks. No material was received for 12 cases and four further cases proved to have been incorrectly registered as sarcomas on scrutiny of the original pathology report and so material was not processed. Diagnosis of sarcoma had been made on clinical grounds in 19 individuals. Of the 429 cases processed 415 (96.7%) were reviewed by all five pathologists. Because of limited availability of some material, six (1.4%) were seen by only four pathologists, six (1.4%) by three and the remaining two (0.5%) by two pathologists.

Final diagnoses were arrived at by the panel for 421 of the 429 reviewed cases. The broad histological categories of the final diagnoses were as follows: sarcoma, specified histology 279; sarcoma NOS 36; connective tissue tumour of borderline malignancy 12; benign connective tissue tumour 13; carcinoma 29; other specified malignant tumour 20; malignant tumour NOS 23; non-neoplastic condition nine; and non-diagnosable material eight. Of the five cases included because of possible uncertainty in diagnosis the original diagnoses of giant cell tumour of bone and atypical fibroxanthoma were confirmed, the case of haemangiopericytoma could only be defined as a borderline soft tissue tumour and no tumour tissue was obtained for the case of haemangioendothelioma. Hence none of these five cases contributed to the final total of sarcomas. For the 13 cases not registered as sarcomas but included in the study because of mention of sarcoma on the registration form, only two had a final diagnosis of sarcoma, one previously diagnosed as malignant spindle cell tumour and another as transitional cell carcinoma.

Of the 421 cases with agreed final diagnoses, 315 were confirmed as sarcomas by the panel, 58 of bone and 257 of soft tissue. Table II lists the final panel diagnoses for the 315 sarcoma cases. A more detailed description of incidence of histological sub-types will be presented elsewhere. Histological diagnoses for the 93 cases originally registered as sarcomas but where sarcoma was not confirmed are shown in Table III.

The clinically diagnosed cases consisted of nine oesteosarcoma, nine sarcoma not otherwise specified and one chordoma. Because of this distribution the original cancer registry diagnoses in the sample of cases for which material was reviewed was markedly different from the diagnoses of the sample for which no review was possible (comparing proportions of osteosarcoma, sarcoma NOS and other specified sarcoma $\chi^2_2 = 39.79$, $P = 0.00001$). This difference should be borne in mind when comparisons between Table I and Table II are made.

**Table II** Final diagnoses for cases diagnosed by the panel as sarcomas

| *Histology* | *Total (1982–84)* |
|---|---|
| *Soft tissue tumours* | |
| Leiomyosarcoma | |
| Gastro-intestinal tract | 14 |
| Female genital tract | 20 |
| Soft tissue | 34 |
| Other sites | 4 |
| Malignant fibrous histiocytoma | 49 |
| Sarcoma NOS | 35 |
| Liposarcoma | 21 |
| Malignant peripheral nerve sheath tumour | 14 |
| Rhabdomyosarcoma | 11 |
| Haemangiosarcoma | 10 |
| Endometrial stromal sarcoma | 9 |
| Synovial sarcoma | 5 |
| Other specified soft tissue sarcoma | 31 |
| Total soft tissue tumours | 257 |
| *Bone tumours* | |
| Osteosarcoma | 24 |
| Chondrosarcoma | 22 |
| Ewing's tumour | 8 |
| Malignant fibrous histiocytoma | 1 |
| Haemangiosarcoma | 1 |
| Chordoma | 1 |
| Sarcoma NOS | 1 |
| Total bone tumours | 58 |
| Total sarcomas | 315 |

**Table III** Final diagnoses for cases diagnosed by the panel as non-sarcomas

| *Histology* | *Total (1982–84)* |
|---|---|
| *Malignant tumours* | |
| Carcinoma NOS | 11 |
| Squamous cell carcinoma | 8 |
| Clear cell adenocarcinoma | 2 |
| Hepatocellular carcinoma | 1 |
| Renal cell carcinoma | 1 |
| Malignant melanoma | 4 |
| Mixed Müllerian tumour | 8 |
| Yolk sac tumour | 1 |
| Astrocytoma | 1 |
| Lymphoma | 5 |
| Malignant tumour NOS | 21 |
| *Borderline tumours* | |
| Smooth muscle tumour of uncertain malignant potential | 3 |
| Epithelioid leiomyoma | 1 |
| Cellular leiomyoma | 1 |
| Haemangiopericytoma NOS | 3 |
| *Benign tumours* | |
| Fibrous histiocytoma | 1 |
| Lipoma | 2 |
| Leiomyoma | 3 |
| Bizarre leiomyoma | 2 |
| Capillary haemangioma | 1 |
| Juxtacortical chondroma | 1 |
| Neurofibroma | 2 |
| Benign soft tissue tumour NOS | 1 |
| *Non-neoplastic conditions* | |
| Proliferative myositis | 1 |
| Fibrous dysplasia | 1 |
| Fibromatosis NOS | 4 |
| Nodular fasciitis | 1 |
| Non-neoplastic NOS | 1 |
| Normal cellular morphology | 1 |
| Total | 93 |

*Agreement between original cancer registry diagnoses and final panel diagnoses*

Material was obtained and reviewed for 413 of the 450 cases originally registered with the cancer registry as sarcomas. The panel agreed that 313 of the 413 (76%) were sarcomas but disagreed with the diagnosis of sarcoma in 72 cases (17%). Twenty-one cases (5%) could be classified only as malignant tumour NOS and the panel was unable to come to any diagnosis in seven cases (2%).

Specific histological sub-types of sarcoma has previously been defined for 338 of the 413 cases originally registered as sarcomas and for which material was reviewed. The panel agreed with the sub-type in 178 cases (53%), disagreed in 57 cases (17%) and were unable to specify a sub-type in 24 (7%). Sixty (18%) were regarded as tumours other than sarcomas, and 12 (4%) classified only as malignant tumour NOS. No diagnosis was given for seven cases (2%).

Cancer registry diagnosis had been non-specific for the remaining 75 cases. Of these the panel defined a specific histological sub-type for 42 (56%), agreed with the original diagnosis of sarcoma NOS in 12 (16%), diagnosed the tumour as other than sarcoma in 12 cases (16%) and were able to diagnose only as malignant tumour NOS in nine cases (12%).

Because of the differing biological and clinical features of sarcomas of diverse sites, degree of agreement was also separately measured for bone sarcomas and for soft tissue sarcomas of visceral and non-visceral origin. Agreement on diagnosis of sarcoma between cancer registry and panel was 87% for bone tumours, 61% for visceral tumours and 78% for non-visceral tumours. Agreement on sub-type was 78%, 52% and 45% for bone, visceral and non-visceral tumours respectively. Of those cases with a cancer registry diagnosis of sarcoma NOS, 67% of bone (2/3), 40% of visceral tumours and 61% of non-visceral tumours were given specific sub-types by the panel. The differences between sites were significant for diagnosis of sarcoma and diagnosis of sub-type ($P = 0.001$ in each case).

In summary, of the 315 cases with a final diagnosis of sarcoma (313 originally selected as sarcomas and two selected as possible sarcomas) the cancer registry and panel agreed on histological sub-type in 178 cases (57%) and disagreed on sub-type in 57 (18%). In 24 cases (8%) the cancer registry specified a sub-type but a non-specific diagnosis was given by the panel and in 42 (13%) the panel was able to specify a sub-type where the original diagnosis had been non-specific. In 12 cases (4%) the original non-specific diagnosis was upheld by the panel and in two cases (1%) the panel specified a particular type of sarcoma where the original registered diagnosis had been of malignant tumour other than sarcoma.

Because of the small numbers in each category of sarcoma, statistical comparisons between original cancer registry diagnosis and final panel diagnosis were made for only five histological sub-types. Measurement of degree of agreement using Cohen's kappa statistic gave values of 0.38 for diagnosis of malignant fibrous histiocytoma, 0.55 for leiomyosarcoma, 0.58 for liposarcoma, 0.73 for osteosarcoma and 0.81 for chondrosarcoma. The relatively low values for malignant fibrous histiocytoma, leiomysarcoma and liposarcoma indicate a wide discrepancy between cases originally registered with those diagnoses and the confirmation of that diagnosis by the panel, and also that a large proportion of cases with a final diagnosis of these types had originally been diagnosed as other sub-types of sarcoma. Table IV gives further information on reclassification of tumour types for the most commonly diagnosed tumours in the series.

**Table IV** Re-classification of tumour types (all sites combined)

| Original diagnosis | Total number reviewed | Final diagnosis | | | | | | | | | | | | | | | | | |
|---|---|---|---|---|---|---|---|---|---|---|---|---|---|---|---|---|---|---|---|
| | | Sarcoma NOS | Leio | MFH | Lipo | Fibro | RMS | Haemgio | Endo stro | Synovial | MPNST | Osteo | Chondro | Ewing's | Other specified sarcoma | Other malignant tumour | Malignant tumour NOS | Benign/ borderline | Non-neoplastic |
| Sarcoma NOS | 73 | **11** | 10 | 8 | 2 | 2 | 0 | 1 | 2 | 1 | 5 | 4 | 1 | 0 | 5 | 11 | 9 | 1 | 0 |
| Leiomyosarcoma | 66 | 1 | **43** | 2 | 0 | 0 | 0 | 0 | 1 | 0 | 1 | 0 | 0 | 0 | 0 | 6 | 1 | 10 | 1 |
| Malignant fibrous histiocytoma | 52 | 6 | 10 | **23** | 1 | 0 | 1 | 0 | 0 | 0 | 0 | 0 | 0 | 0 | 1 | 4 | 3 | 2 | 1 |
| Liposarcoma | 35 | 1 | 2 | 4 | **17** | 0 | 0 | 0 | 0 | 0 | 0 | 0 | 0 | 0 | 1 | 6 | 1 | 3 | 0 |
| Fibrosarcoma | 28 | 5 | 4 | 5 | 0 | **2** | 0 | 0 | 0 | 1 | 2 | 1 | 2 | 0 | 1 | 1 | 2 | 1 | 1 |
| Rhabdomyosarcoma | 25 | 5 | 1 | 5 | 1 | 0 | **9** | 0 | 0 | 0 | 0 | 0 | 0 | 0 | 1 | 1 | 2 | 0 | 0 |
| Haemangiosarcoma | 14 | 2 | 0 | 0 | 0 | 0 | 0 | **9** | 0 | 0 | 0 | 0 | 0 | 0 | 0 | 2 | 0 | 1 | 0 |
| Endometrial stromal sarcoma | 10 | 1 | 0 | 1 | 0 | 0 | 0 | 0 | **7** | 0 | 0 | 0 | 0 | 0 | 0 | 1 | 0 | 0 | 0 |
| Synovial sarcoma | 4 | 1 | 0 | 0 | 0 | 0 | 0 | 0 | 0 | **3** | 0 | 0 | 0 | 0 | 0 | 0 | 0 | 0 | 0 |
| Neurofibrosarcoma | 10 | 0 | 1 | 0 | 0 | 0 | 1 | 0 | 0 | 0 | **6** | 0 | 0 | 0 | 0 | 1 | 0 | 1 | 0 |
| Osteosarcoma | 28 | 1 | 0 | 0 | 0 | 0 | 0 | 0 | 0 | 0 | 0 | **21** | 1 | 1 | 0 | 2 | 1 | 0 | 1 |
| Chondrosarcoma | 30 | 0 | 0 | 0 | 0 | 0 | 0 | 0 | 0 | 0 | 0 | 2 | **24** | 0 | 0 | 2 | 0 | 1 | 1 |
| Ewing's tumour | 9 | 0 | 0 | 0 | 0 | 0 | 0 | 0 | 0 | 0 | 0 | 0 | 0 | **9** | 0 | 0 | 0 | 0 | 0 |

### *Agreement between individual pathologists and final panel diagnosis*

Table V gives some measures of agreement in diagnosis between individual pathologists (arranged in random order), in relation to final panel diagnoses. Type A and type B agreements were accepted for this comparison. For the 315 cases agreed upon as sarcomas by the panel, pathologist 1, for example, saw 313 cases and diagnosed 311 of the 313 specimens as neoplastic, considered 304 to be malignant, thought that 303 were sarcomas and specified a specific sub-type of sarcoma which agreed with the final panel diagnosis in 223 cases. This pathologist reported on all 50 cases finally diagnosed as malignant fibrous histiocytoma and agreed with that diagnosis in 43 instances.

### Discussion

Histological material was obtained for 96% (429/449) of the 468 cases entered in this study and for whom previous histopathological diagnosis had been recorded. Of these, 96.7% were reviewed by all five pathologists on the panel and final diagnoses were arrived at for 98% (421) cases. Hence the successful review of almost the whole population of sarcomas diagnosed in North West England over the 3-year period 1982–84 (excluding 19 cases clinically diagnosed) enabled a reliable assessment to be made of the results of histopathological peer review.

Material was reviewed for 413 of the 450 cases originally registered as sarcomas with the Regional Cancer Registry but in only 313 (76%) of cases could the diagnosis of sarcoma be confirmed by the panel. Furthermore, where a specific sub-type of sarcoma had previously been specified the panel agreed with the sub-type in only 53% of cases, although they were able to specify a sub-type in 56% of previously non-specified cases.

These results are, in general, consistent with those of similar studies. Presant *et al.* (1986) reported the review of specimens from 216 consecutive patients with bone or soft tissue sarcomas entered into trials conducted by the Southeastern Cancer Study Group (SEG). Most cases were reviewed by one or two pathologists in addition to the original reviewer, and there was agreement between primary reviewer and panel in 66% cases. In 27% there was disagreement over sub-type and 6% cases were considered not to be sarcomas. Reports of a similar panel review of 130 cases of disseminated soft tissue sarcoma by the Southwest Oncology Group (Baker *et al.*, 1978) showed total agreement on sub-type in 62% cases, disagreement in 32% and a non-sarcoma diagnosis in 7%. Review of 240 patients aged 15–70 years with localised high grade soft tissue sarcomas by the Scandinavian Sarcoma Group (Alvegård *et al.*, 1989) also resulted in change of sub-type in 25% cases and in 5% of cases being rejected as non-sarcomas.

Change in diagnosis from sarcoma to other tumours in the three studies described above was consistent at 5–7% cases. One of the striking results of this study was the much larger proportion of cases (22%) where such a change was made. In 15% the change was to other types of malignant tumour or malignant tumour NOS, in 3% to benign tumours, in 2% to borderline tumours and in 2% to non-neoplastic conditions (the remaining 2% were unclassified). The reason for this high level of reclassification probably lies in the fact that this was a population-based series whereas previous studies were based upon cases referred for trials of adjuvant therapy. Hence many of the cases in this series would not have been referred to specialist centres for treatment because, for example, they were very elderly, had advanced disease at diagnosis, or were only diagnosed at post-mortem examination. In addition because of the wide geographical spread of the cases, the original specimens had been reported by a large number of different pathologists, some of whom would see very few cases of sarcoma each year.

Because of small numbers, statistical measures of agree-

**Table V** Agreement between individual pathologists and final panel diagnosis for cases diagnosed as sarcomas (all sites combined)

| *Diagnosis (no. of cases)* | *Pathologist 1* *No.* | *%* | *2* *No.* | *%* | *3* *No.* | *%* | *4* *No.* | *%* | *5* *No.* | *%* |
|---|---|---|---|---|---|---|---|---|---|---|
| Neoplastic condition (315) | 311/313 | (99.4) | 314/315 | (99.7) | 308/311 | (99.0) | 308/310 | (99.4) | 311/312 | (99.7) |
| Malignant tumour (315) | 304/313 | (97.1) | 306/315 | (97.1) | 299/311 | (96.1) | 297/310 | (95.8) | 304/312 | (97.4) |
| Sarcoma (315) | 303/313 | (96.8) | 311/315 | (98.7) | 298/311 | (95.8) | 296/310 | (95.5) | 297/312 | (95.2) |
| Sarcoma sub-type where specified (279) | 223/277 | (80.5) | 238/278 | (85.6) | 184/275 | (66.9) | 178/274 | (65.0) | 227/276 | (82.2) |
| Malignant fibrous histiocytoma (50) | 43/50 | (86.0) | 41/50 | (82.0) | 21/50 | (42.0) | 30/50 | (60.0) | 40/50 | (80.0) |
| Leiomyosarcoma (72) | 57/72 | (79.2) | 62/72 | (86.1) | 53/71 | (74.6) | 40/71 | (56.3) | 64/72 | (88.9) |
| Liposarcoma (21) | 16/20 | (80.0) | 19/21 | (90.5) | 18/21 | (85.7) | 16/20 | (80.0) | 18/20 | (90.0) |
| Osteosarcoma (28) | 21/27 | (77.8) | 24/27 | (88.9) | 18/27 | (66.7) | 25/28 | (89.3) | 21/27 | (77.8) |
| Chondrosarcoma (28) | 24/28 | (85.7) | 27/28 | (96.4) | 26/28 | (92.9) | 24/28 | (85.7) | 24/27 | (88.9) |

ment between the original diagnosis and the panel diagnosis were made for only five tumour types. Degree of agreement was high for osteosarcoma and chondrosarcoma and low for the two sub-types most frequently diagnosed i.e. malignant fibrous histiocytoma (MFH) and leiomyosarcoma.

MFH was the most commonly diagnosed sarcoma in adults during the last two decades but evidence is now accumulating that its most common variant, the pleomorphic storiform type, is very heterogeneous and may have been used as a diagnosis of convenience for a variety of neoplasms in which no specific line of differentiation could be determined (Fletcher, 1990). In only 23 of the 52 cases of MFH in this series was the diagnosis confirmed; ten were reclassified as leiomyosarcoma and six as sarcoma NOS, perhaps reflecting the over-utilisation of this non-specific diagnosis.

Although almost two-thirds (43/66) of the leiomyosarcomas were confirmed as such, six were reclassified as malignant tumours other than sarcomas and ten as benign or borderline tumours. Leiomyosarcomas formed the single largest group of sarcomas of visceral sites in this series and degree of agreement for this sub-type was lower for tumours of female genital tract (Cohen's kappa = 0.32) than for those of gastro-intestinal tract (0.44), of other soft tissue sites (0.46) and of other visceral sites (0.63). Almost 50% of leiomyosarcomas of female genital tract were in fact reclassified, seven as leiomyomas or as smooth muscle tumours of uncertain malignant potential, three as Müllerian mixed tumours and one as endometrial stromal sarcoma. Some difficulty however, was experienced in defining degree of malignancy in these tumours particularly where sampling of tumour was felt to be inadequate.

Diagnosis of osteosarcoma and chondrosarcoma was much more consistent as would be expected and, in spite of its non-specificity in appearance, there was a high degree of agreement for Ewing's tumour with all nine cases seen by the panel being confirmed as such.

Diagnosis of liposarcoma was very variable, with only 17 of 35 confirmed. The majority of cases of rhabdomyosarcoma (RMS) were also reclassified (16 out of 25) on review. This is in contrast with the findings of Newton *et al.* (1988) who reported 94% agreement between the review committee and institutional pathologists in the diagnosis of RMS, although much less agreement on sub-type. This discrepancy is again probably related to the type of patient entered into the study.

Perhaps the most striking reclassification was of fibrosarcoma with only two out of 28 cases confirmed. Five were reclassified as MFH, four as leiomyosarcoma, five as sarcoma NOS and the rest were spread over a variety of different types. Fibrosarcoma was the most common sarcoma diagnosis made 30 years ago but has perhaps been the most affected by changing diagnostic criteria until at the present time the diagnosis is rarely made.

The study demonstrated a high degree of agreement on the diagnosis of sarcoma between individual pathologists on the panel, although the diagnosis of sub-type was much less consistent. Because, however, Type A and Type B agreements were accepted for sub-type comparisons, the bias was in favour of those pathologists who submitted more than two differential diagnoses, rather than those whose diagnoses were more precise. Differences between pathologists may reflect their experience in terms of numbers of sarcomas reviewed prior to taking part in the study and also their differing expertise in relation to bone or soft tissue tumours.

While the degree of disagreement between original diagnosis and final panel diagnosis, and between individual pathologists and the final panel diagnosis is disturbing, it should perhaps be noted that while being a consistent finding for histopathological studies of sarcomas, such reclassification is much less common for most other types of malignant disease (Whitehead *et al.*, 1984).

Variation between the opinions of the original pathologist and the panel members is inevitable. In some cases the panel may have received unrepresentative samples of the tumour for review, especially where there was histological variability between areas of tumour, and in other cases inadequate provision of different samples of the same tumour could have resulted in the mis-diagnosis of grade of malignancy. Nevertheless it is surprising that, in spite of seeing relatively large numbers of sarcomas over and above normal workload, such variation in diagnosis between panel members continued to occur and although no formal assessment of converging agreement with time was made, the general impression was that this did not take place. This impression is consistent with that of Presant *et al.* (1986) who found no improvement in frequency of agreement in the course of the SEG study, in spite of educational workshops. The only conclusion which can be drawn from this is that second opinion is of vital importance in cases of presumed sarcomas, particularly for those cases e.g. MFH where concordance in diagnosis appears to be low. Review is essential so that correct treatment is selected for those patients who are almost certain to have sarcomas and so that inappropriate therapy is not given to cases who do not have sarcomas, or who have only benign or boderline or non-neoplastic conditions.

We are grateful to the North West Regional Cancer Registry for the provision of data relating to registration of sarcomas. We would also like to thank the many pathologists who provided material for the study including S. Banik, R.W. Blewitt, W.G. Brown, C.H. Buckley, J. Burns, A.B. Colclough, K.S. Daber, A.S. Day, D.M.H. De Krester, S. Dutt, A.R. Evans, G. Garrett, R. Gillett, J.R. Goepel, I. Gupta, B.N.A. Hamid, D.S. Harry, P.S. Hasleton, C.K. Heffernan, J.R. Helliwell, S.S. Hom-Choudhury, A.C. Hunt, N.N. Jaswon, A.R. Mainwaring, H.B. Marsden, J.A. Morris, H.M. Myat, W.G. Owen, N.L. Reeve, W.H. Richmond, C.M. Starkie, V. Tagore, W.H. Taylor, E.G.F. Tinsley, J.M. Torry, D.M. Vickers, S. Wells, J.S. Whittaker, G. Williams, H.D. Zakhour.

We are particularly grateful to Ewa Dale who scrutinised the cancer registrations and coordinated the receipt and despatch of material, and to Delyth Elliott who typed the manuscript.

This work was supported by the Cancer Research Campaign.

### References

ALVEGÅRD, T.A. & BERG, N.O. for the Sandinavian Sarcoma Group (1989). Histopathology peer review of high-grade soft tissue sarcoma: The Scandinavian Sarcoma Group Experience. *J. Clin. Oncol.*, **7**, 1845.

BAKER, L.H. & BENJAMIN, R.S. (1978). Histologic frequency of disseminated soft tissue sarcomas in adults. *Proc. Am. Soc. Clin. Oncol.*, **19**, 324 (Abstr.).

ENZINGER, F.M. & WEISS, S.W. 2nd Edn (1988). *Soft Tissue Tumors.* C.V. Mosby Co.: St. Louis.

FLETCHER, C.D.M. (1990). Recent advances in the pathology of soft tissue tumours. *Diagn. Oncol.*, **1**, 5.

FLEISS, J.L. (1981). *Statistical Methods for Rates and Proportions.* J. Wiley, New York. p. 217.

MUIR, C., WATERHOUSE, J., MACK, T., POWELL, J. & WHELAN, S. (1987). *Cancer Incidence in Five Continents*, Volume V. IARC Scientific Publication No. 88, IARC, Lyon.

NEWTON, W.A. Jr, SOULE, E.H., HAMOUDI, A.B. & 4 others (1988). Histopathology of childhood sarcomas, Intergroup Rhabdomyosarcoma Studies I and II: clinicopathologic correlation. *J. Clin. Oncol.*, **6**, 67.

NORUSIS, M.J. (1989). *SPSS/PC+™ V3.0 Base Manual.* SPSS Inc.: Chicago.

PRESANT, C.A., RUSSELL, W.O., ALEXANDER, R.W. & FU, Y.S. (1986). Soft-tissue and bone sarcoma histopathology peer review: the frequency of disagreement in diagnosis and the need for second pathology opinions. The Southeastern Cancer Study Group Experience. *J. Clin. Oncol.*, **4**, 1658.

SHIRAKI, M., ENTERLINE, H.T., BROOKS, J.J. & 7 others (1989). Pathologic analysis of advanced adult soft tissue sarcomas, bone sarcomas and mesotheliomas. The Eastern Cooperative Oncology Group (ECOG) experience. *Cancer*, **64**, 484.

WHITEHEAD, M.E., FITZWATER, J.E., LINDLEY, S.K., KERN, S.B., ULIRSCH, R.C. & WINECOFF, W.F. (1984). Quality assurance of histopathologic diagnoses: a prospective audit of three thousand cases. *Am. J. Clin. Pathol.*, **81**, 487.

WORLD HEALTH ORGANISATION (1976). *ICD-O: International Classification of Diseases for Oncology.* World Health Organisation: Geneva.